# Right Mini-Thoracotomy for Mitral and Tricuspid Repair in Abdominal Situs Inversus

**DOI:** 10.1055/a-2938-6270

**Published:** 2026-08-31

**Authors:** Yuka Kaijima, Tomoyuki Suzuki, Payam Akhyari, Yukiharu Sugimura

**Affiliations:** 1 Department of Thoracic and Cardiovascular SurgeryWest German Heart and Vascular Center, University Hospital EssenEssenGermany; 2 Keio University School of MedicineShinjuku–kuTokyoJapan

**Keywords:** cardiovascular surgery, heart disease, heart valve surgery, minimally invasive surgery, mitral valve surgery

## Abstract

**Background:**

Heterotaxy with abdominal situs inversus complicates cardiac surgery due to mirror-image anatomy and associated venous anomalies.

**Case Description:**

A 61-year-old woman with heterotaxy, abdominal situs inversus, atrial fibrillation, and prior dual-chamber cardiac pacemaker implantation presented with New York Heart Association class III dyspnea and severe secondary mitral and tricuspid regurgitation. Preoperative imaging revealed a narrow left-sided inferior vena cava and persistent left superior vena cava. Minimally invasive multivalvular repair via right minithoracotomy was successfully performed using dual-site venous drainage.

**Conclusion:**

Tailored venous drainage based on detailed preoperative imaging enables safe minimally invasive surgery in patients with complex venous anatomy.

## Introduction

Heterotaxy is a rare congenital condition characterized by an abnormal spatial arrangement of the thoracic and abdominal organs and is frequently associated with complex systemic venous anomalies. 1 These anatomical variations may complicate cardiac surgery, particularly with regard to anatomical orientation, cannulation, and establishment of adequate venous drainage for cardiopulmonary bypass (CPB). Median sternotomy has traditionally been considered the standard approach in such patients because it facilitates anatomical orientation, exposure, and cannulation.

Minimally invasive cardiac surgery (MICS) has gained increasing acceptance because of reduced surgical trauma and faster postoperative recovery. 2 However, its application in patients with complex systemic venous anomalies remains challenging, particularly with regard to establishing adequate venous drainage for CPB. 3 4

We report a case of minimally invasive multivalvular repair in a patient with heterotaxy, abdominal situs inversus, and complex systemic venous anatomy, highlighting a planned dual-site venous drainage strategy.

## Case Description

**Video 2** Intraoperative video demonstrating endoscopy-assisted minimally invasive mitral and tricuspid valve repair via right minithoracotomy in a patient with heterotaxy, abdominal situs inversus, and complex systemic venous anomalies. After establishment of the planned dual-site venous drainage strategy, mitral valve repair was performed using a 30-mm Carpentier-Edwards Physio II annuloplasty ring. Tricuspid valve repair was subsequently performed using a 30-mm Carpentier-Edwards Physio Tricuspid annuloplasty ring. Direct pump suction placed in the coronary sinus provided a bloodless operative field during tricuspid valve repair.


**Fig. 1:**
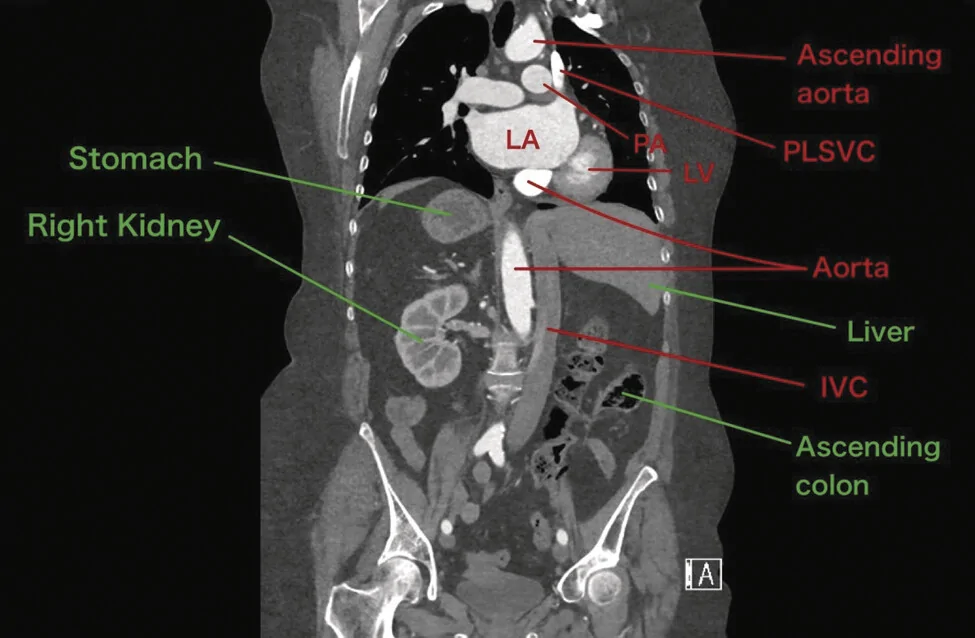
Heterotaxy and systemic venous anatomy (coronal view). Coronal contrast-enhanced computed tomography demonstrating heterotaxy with a mirror-image arrangement of the thoracoabdominal organs. Note the left-sided IVC and right-sided abdominal aorta, confirming the reversal of normal lateralization. A PLSVC is also visualized draining into a dilated coronary sinus. IVC, inferior vena cava; LA, left atrium; LV, left ventricle; PA, pulmonary artery; PLSVC, persistent left superior vena cava.

**Fig. 2:**
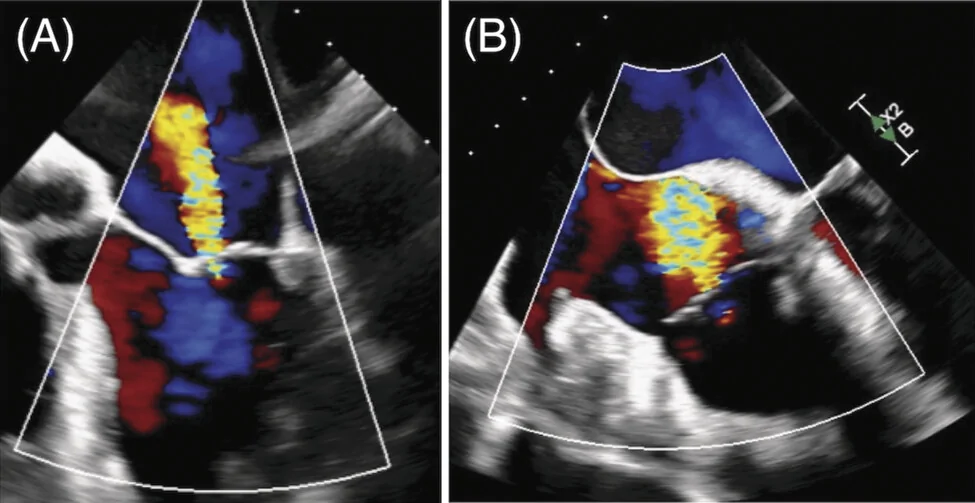
Preoperative mitral and tricuspid regurgitation. Transesophageal echocardiography demonstrating severe mitral regurgitation ( **A** ) and severe tricuspid regurgitation ( **B** ).

**Fig. 3:**
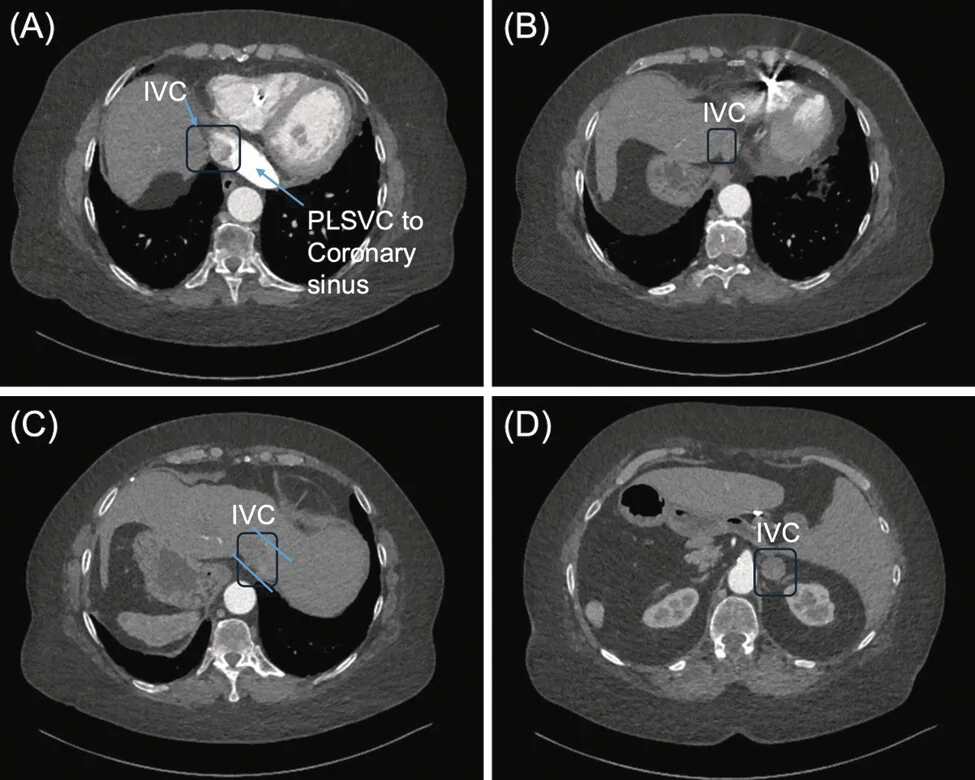
Axial CT sequence demonstrating the tortuous course of the left-sided IVC. A series of four axial contrast-enhanced CT slices from the midcardiac level (upper panels) to the hepatic level (lower panels). **(A–D)** Sequential images demonstrate the sinuous and angulated course of the left-sided IVC, as it ascends anterior and lateral to the vertebral bodies. The relationship between the IVC and the aorta is reversed compared with normal anatomy. This marked tortuosity and narrow caliber of the IVC (arrows) explain the resistance encountered during the advancement of the femoral venous cannula, necessitating a modified cannulation strategy. CT, computed tomography; IVC, inferior vena cava; PLSVC, persistent left superior vena cava.

A 61-year-old woman with heterotaxy characterized by abdominal situs inversus ( Fig. 1 ) and thoracic situs solitus was referred to our institution because of progressive dyspnea corresponding to New York Heart Association functional class III. Her medical history included dual-chamber cardiac pacemaker implantation for symptomatic sinus bradycardia with clinically relevant sinus pauses, paroxysmal atrial fibrillation, arterial hypertension, Crohn's disease, and exogenous allergic alveolitis with restrictive pulmonary impairment. Preoperative transesophageal echocardiography demonstrated severe mitral regurgitation secondary to left atrial enlargement, consistent with Carpentier type I dysfunction, and severe tricuspid regurgitation ( Fig. 2 ). Coronary angiography showed no significant coronary artery disease. Computed tomography demonstrated heterotaxy with abdominal situs inversus and thoracic situs solitus, together with a left-sided inferior vena cava and a persistent left superior vena cava (PLSVC; Fig. 3 ).

Catheter-based venography, which had been performed prior to pacemaker implantation, confirmed continuity of the inferior vena cava and demonstrated its markedly tortuous intra-abdominal course. Venography also confirmed the presence of both a right superior vena cava and PLSVC. No definite evidence of azygos or hemiazygos continuation into the PLSVC was identified ( Video 1 , available in online version only).

Following interdisciplinary Heart Team discussion, one-stage minimally invasive surgery via right minithoracotomy was planned, including mitral valve repair (MVr), tricuspid valve repair (TVr), patent foramen ovale (PFO) closure, and left atrial appendage (LAA) closure.

Surgery was performed under CPB with femoral arterial cannulation. Based on the preoperative recognition of complex systemic venous anatomy, a dual-site venous drainage strategy was planned in advance. Femoral venous cannulation was performed; however, advancement of the venous cannula was technically challenging because of the tortuous intra-abdominal course of the left-sided inferior vena cava. Under fluoroscopic guidance, the cannula was eventually advanced to the level of the diaphragm. A second venous cannula was inserted directly into the right atrium through the right minithoracotomy, thereby completing the planned dual-site venous drainage strategy. After adequate CPB flow was established, MVr and TVr were performed under endoscopic assistance ( Video 2 , available in the online version only). MVr was performed with implantation of a 30-mm Carpentier-Edwards Physio II annuloplasty ring (Edwards Lifesciences LLC). During TVr, additional pump suction was placed in the coronary sinus to improve local venous decompression and provide a bloodless operative field. This suction was used as an adjunct for operative exposure and was not part of the principal venous drainage strategy.

**Fig. 4:**
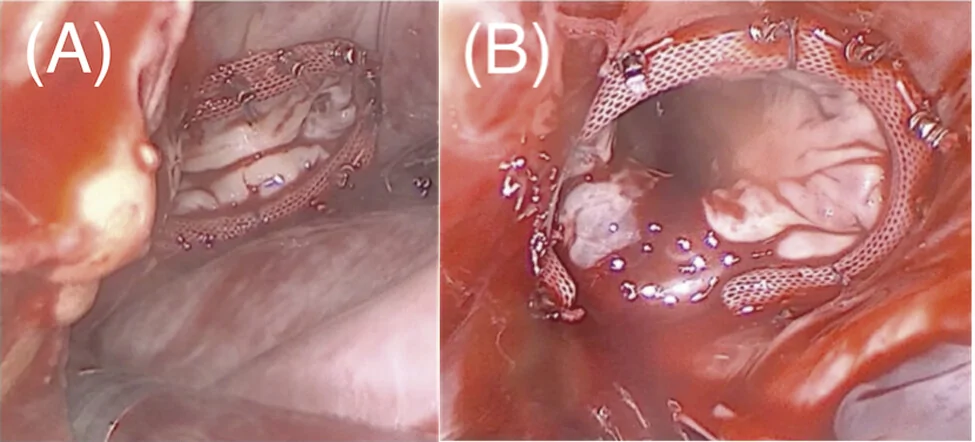
Intraoperative view of mitral and tricuspid valve repair. Intraoperative view through right minithoracotomy demonstrating mitral valve repair ( **A** ) and tricuspid valve repair ( **B** ).

After all annular sutures for TVr had been placed, the aortic cross-clamp was released before ring implantation. TVr was subsequently completed with implantation of a 30-mm Carpentier-Edwards Physio Tricuspid annuloplasty ring (Edwards Lifesciences LLC) ( Fig. 4 ). PFO closure was performed using a direct suture technique. Epicardial LAA exclusion was performed by using an AtriClip device (AtriCure, Inc). The total operative time was 308 minutes, and the aortic cross-clamp time was 76 minutes.

The patient was extubated on the day of surgery and transferred to the cardiac intermediate care unit on postoperative day 1. On postoperative day 5, respiratory failure required reintubation and readmission to the intensive care unit. On postoperative day 13, she was transferred to an affiliated pulmonary specialty hospital for respiratory rehabilitation. Survival was confirmed at 30 days postoperatively.

## Discussion

**Video 1** Preoperative catheter-based venography demonstrating continuity and the markedly tortuous intra-abdominal course of the left-sided inferior vena cava, as well as the presence of a right superior vena cava and a persistent left superior vena cava. No definite azygos or hemiazygos continuation into the persistent left superior vena cava was identified. The catheter-based venographic examination had been performed before implantation of the DDD pacemaker. Therefore, no transvenous pacemaker leads were present at the time of venography. DDD, dual-chamber pacemaker.


The key technical message of this case is that complex systemic venous anomalies can significantly compromise conventional venous drainage strategies during MICS.

PLSVC and other venous anomalies require careful modification of standard cannulation techniques. Previous reports have emphasized the importance of individualized venous drainage strategies tailored to anatomical variations. 3 4

In the present case, preoperative imaging demonstrated a combination of PLSVC, left-sided inferior vena cava, and transvenous pacemaker leads. These anatomical features directly impacted CPB strategy. Femoral venous cannulation alone was insufficient because the cannula could not be advanced adequately due to the angulated course of the inferior vena cava, resulting in inadequate venous return. This limitation should be considered preoperatively when detailed imaging reveals complex systemic venous anatomy, underscoring the importance of meticulous anatomical assessment before MICS.

To overcome this issue, dual-site venous drainage was established by adding direct right atrial cannulation. In addition, direct right atrial pump suction was used to further improve venous decompression and stabilize CPB flow. This modified strategy proved effective in this case, allowing completion of the procedure without conversion to median sternotomy.

Minimally invasive mitral valve surgery has been associated with reduced surgical trauma and faster recovery compared with conventional sternotomy. 2 5 6 However, these advantages depend on the ability to maintain stable CPB conditions. Therefore, in patients with complex venous anatomy, procedural success relies not only on surgical expertise but also on flexible adaptation of venous drainage strategies.

In addition, concomitant LAA closure may reduce thromboembolic risk in patients with atrial fibrillation undergoing cardiac surgery. 7 In the present case, no concomitant ablation procedure was performed given the patient's long-standing atrial fibrillation and marked left atrial enlargement; LAA closure alone was undertaken.

This case highlights the importance of detailed preoperative assessment and intraoperative flexibility. Surgeons should anticipate the need for alternative or adjunctive venous drainage strategies in patients with complex systemic venous anatomy, particularly when venous anomalies coexist with intracardiac devices.

In conclusion, individualized venous drainage strategies based on detailed anatomical assessment are essential for safe MICS in patients with complex venous anatomy.

## Declaration of GenAI Use

The authors used ChatGPT (OpenAI) during the preparation of this manuscript for language editing and improvement of readability. The authors reviewed and revised all output and take full responsibility and accountability for the content of the article.
